# In-depth investigation of the point mutation pattern of HIV-1

**DOI:** 10.3389/fcimb.2022.1033481

**Published:** 2022-11-15

**Authors:** Nan Sun, Stephen S.-T. Yau

**Affiliations:** ^1^ Department of Mathematical Sciences, Tsinghua University, Beijing, China; ^2^ Yanqi Lake Beijing Institute of Mathematical Sciences and Applications, Beijing, China

**Keywords:** mutation, HIV, SNP, natural vector, nucleotide distribution

## Abstract

Mutations may produce highly transmissible and damaging HIV variants, which increase the genetic diversity, and pose a challenge to develop vaccines. Therefore, it is of great significance to understand how mutations drive the virulence of HIV. Based on the 11897 reliable genomes of HIV-1 retrieved from HIV sequence Database, we analyze the 12 types of point mutation (A>C, A>G, A>T, C>A, C>G, C>T, G>A, G>C, G>T, T>A, T>C, T>G) from multiple statistical perspectives for the first time. The global/geographical location/subtype/k-mer analysis results report that A>G, G>A, C>T and T>C account for nearly 64% among all SNPs, which suggest that APOBEC-editing and ADAR-editing may play an important role in HIV-1 infectivity. Time analysis shows that most genomes with abnormal mutation numbers comes from African countries. Finally, we use natural vector method to check the k-mer distribution changing patterns in the genome, and find that there is an important substitution pattern between nucleotides A and G, and 2-mer CG may have a significant impact on viral infectivity. This paper provides an insight into the single mutation of HIV-1 by using the latest data in the HIV sequence Database.

## 1 Introduction

Human immunodeficiency virus (HIV) is a virus that attacks the host’s immune system. It makes people more vulnerable to other diseases (Weiss, 1993). Without treatment, the two types of HIV (HIV-1 and HIV-2) infection will cause acquired immunodeficiency syndrome (AIDS). HIV-1 is more infective than HIV-2 and is the main cause of global HIV infection (Gilbert et al., 2003). According to the HIV statistics data of UNAIDS (Joint United Nations Programme on HIV/AIDS), about 84.2 million people have become infected with HIV and 40.1 million people have died from AIDS-related illnesses since the start of the epidemic. Approximately 1.5 million new HIV infections occurred last year. The COVID-19 pandemic led to disruptions to key HIV treatment and prevention services, millions of people are in danger, the emergency situation makes the effective HIV treatment and tools to prevent, detect and treat infections more significant (UNAIDS, 2022). HIV-1 is a single-stranded, positive-sense RNA retrovirus, its RefSeq (Accession number: NC_001802) length is 9181 bp. HIV has nine major genes (three structural genes: Gag, Pol, Env, two regulatory genes: Tat, Rev, and four accessory genes: Nef, Vif, Vpr, Vpu), encoding nineteen proteins. The mutation of nucleotide sequence encoding protein may affect the ability or efficiency of virus infectivity. Therefore, the identification of mutation type is of great significance for understanding the mechanism of virus infection.

The virus constantly mutates to adapt to the surroundings. Sequence mutation will cause the translocation, insertion, deletion and substitution of bases. The variation of nucleotides may result in codon changes, which may lead to changes in the coding sequences for proteins and their functions (Nishikura, 2010). Many mutations are minor and will not affect the transmission speed or infection degree, even make the virus less infectious. However, some genetic mutations are dangerous. They may cause the surface of the virus to look different from the original virus, leading to immune escape and stronger infectivity (Smyth et al., 2012). On the other hand, mutation is the driving force of species evolution. Evolution is a function of both mutations and selective pressures, eliminating unfavorable mutations and selecting for favorable mutations (Sun et al., 2022a). HIV-1 has high replication rate and is vulnerable to natural selection (Coffin, 1995). The high mutation rate creates genetic diversity and new virus subtypes, which poses a greater challenge to the development of vaccines.

There are many molecular mechanisms that contribute to HIV-1 replication rate such as the DNA repair process associated with the uracil DNA glycosylase (UNG) (Mansky et al., 2000) or the nuclear form of uracil DNA glycosylase (UNG2) (Chen et al., 2004), RNA editing process by deamination. There are two kinds of RNA editing mechanisms by deamination in human cells: APOBEC (apolipoprotein B mRNA editing enzyme, catalytic polypeptide-like) (Zheng et al., 2004) and ADAR (adenosine deaminases acting on double-stranded regions of RNA) (Nishikura, 2016). APOBEC-induced editing involves cytidine deaminase that deaminates a cytidine base into a uridine base (C-to-U mutation). ADAR enzyme deaminates adenine to inosine (A-to-I editing) and causes A-to-G mutation. HIV-1 RNAs contain several double-stranded regions such as the Rev responsive element, trans-activation responsive element and dimerization domain (Phuphuakrat et al., 2008; Doria et al., 2009). Many studies provide evidence that expression levels of the ADAR protein are responsible for A to G mutations in the HIV-1 genome and HIV-1 infection potential (Sharmeen et al., 1991; Clerzius et al., 2009; Weiden et al., 2014). Particularly, APOBEC3G, as a member of APOBEC protein family, induces G-to-A hypermutation in plus-stranded cDNA, which can be readily detected in infected persons; but the virus encodes the Vif protein to counteract APOBEC3G (Malim, 2009). So maybe the interaction of APOBEC3G and ADAR has the influence on the HIV-1 infectivity. In this paper, we hypothesize that gene editing *via* APOBEC and ADAR is a driving force for RNA viral evolution.

We analyze the 12 single-nucleotide polymorphisms (SNPs) types of 11897 HIV-1 complete genomes. SNP is a variation at a single position in a nucleotide sequence among individuals. Looking for SNPs can evaluate the viral infectivity mechanism (Single-nucleotide polymorphism / SNP). We observe that mutations A>G, G>A, C>T and T>C own nearly 64% ratio in non-unique case, which suggests that APOBEC and ADAR editing may drive the virus evolution. In addition, we investigate the distribution of 12 SNP types in different time, countries and subtypes to understand whether mutations have time, geographical location and subtype preferences. We also report the mutation preferences in local regions of the genome, i.e. k-mer. Finally, we use our previously proposed method, k-mer natural vector to identify and validate the mutation preferences in the genome.

## 2 Materials and methods

### 2.1 HIV-1 datasets analyzed

The complete genomes of HIV-1 analyzed in this study are downloaded from HIV sequence Database (https://www.hiv.lanl.gov), including 16844 sequences up to April 8, 2022. The sequences with degenerate bases (i.e. R, Y, M, K, S, W, H, B, V, D, N) and without time or country labels are deleted, and 11897 reliable genomes are retained. The accession number, sampling year, sampling country, subtype of each sequence can be found in the Supplementary Data.

### 2.2 Method overview

The genome sequences are aligned with reference sequence of HIV-1 (GenBank accession number is NC_001802, it sampled in France in 1983 (Barré-Sinoussi et al., 1983; Gallo et al., 1983), and sequence length is 9181 base pairs) using Clustal Omega with default parameters. Then the SNP types (A>C, A>G, A>T, C>A, C>G, C>T, G>A, G>C, G>T, T>A, T>C, T>G) can be counted in the corresponding positions from the aligned genomes. For example, there are 4 sequences totally: RefSeq: ACGTGTGAC, Seq1: CCTTGTGAC, Seq2: TCGTTGTC, Seq3: CCGGGATAAC. The pairwise sequences are aligned as follows:

- The first pairwise sequence alignment:

**Table:** 

Position:	1	2	3	4	5	6	7	8	9
RefSeq:	A	C	G	T	G	T	G	A	C
Seq1	C	C	T	T	G	T	G	A	C

- The second pairwise sequence alignment:

**Table:** 

Position:	1	2	3	4	5	6	7	8	9
RefSeq:	A	C	G	T	G	T	G	A	C
Seq2	T	C	G	T	–	T	G	T	C

- The third pairwise sequence alignment:

**Table:** 

Position:	1	2	3	4	5		6	7	8	9
RefSeq:	A	C	G	T	G	–	T	G	A	C
Seq3	C	C	G	G	G	A	T	A	A	C

We only consider those mutation positions without gaps. The non-unique mutation indicates that the same mutation in different genomes is counted repeatedly, and the unique mutation indicates that the same mutation in different genomes is only counted once. For above three alignments, the non-unique mutation numbers of A1C, A1T, A8T, G3T, T4G, and G7A are 2, 1, 1, 1, 1, 1, respectively, then the non-unique mutation numbers of A>C, A>T, G>T, T>G, and G>A are 2, 2, 1, 1, 1, respectively, and the other 7 SNP types’ number is 0. The unique mutation numbers of A1C, A1T, A8T, G3T, T4G, and G7A are 1, 1, 1, 1, 1, 1, respectively, then the non-unique mutation numbers of A>C, A>T, G>T, T>G, and G>A are 1, 2, 1, 1, 1, respectively, and the other 7 SNP types’ number is 0.

## 3 Results

### 3.1 Single mutation analysis

#### 3.1.1 Global analysis

**Figure 1:**
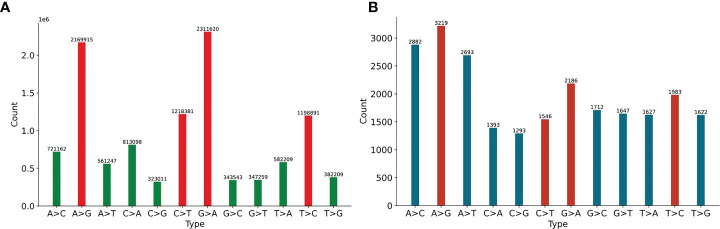
**(A)** The distribution of 12 non-unique mutations counted from the 11897 HIV-1 aligned genomes. The non-unique mutation indicates that the same mutation in different genomes is counted repeatedly. **(B)** The distribution of 12 unique mutations counted from the 11897 HIV-1 aligned genomes. The unique mutation indicates that the same mutation in different genomes is only counted once. The number above the vertical bar is the count of corresponding mutation.

We first analyze 11897 HIV-1 aligned genomes globally. All genomes are aligned with the reference sequence of HIV-1, then the mutations are counted. The proportion of 12 SNP types is shown in **Figure 1**. The distribution of 12 non-unique mutations is displayed in **Figure 1A**. There are 10,972,545 single mutations for 11897 HIV-1 isolates, and each HIV-1 genome has about 922 mutations, which shows that the variation of HIV-1 is diverse (Fischer et al., 2021). The frequency of transition (A>G, G>A, C>T, T>C) is higher than that of transversion (A>C, C>A, A>T, T>A, G>C, C>G, G>T, T>G) (Collins and Jukes, 1994). If the mutation is random, the probability of each mutation is 8.33%, but the four mutations, A-to-G, G-to-A, C-to-T and T-to-C, accounts for 63.87% (=6898807/10972545). One potential explanation is that A>G, G>A, C>T may be driven by the APOBEC and ADAR editing, and these patterns have a significant impact on HIV-1 infectivity. The ratio of G>A is a little higher than that of A>G. It is noticed that ADAR induces A>G mutation and APOBEC3G induce G>A mutation in cDNA, but virus will fight back against the APOBEC3G editing mechanism using encoding protein (Malim, 2009). On the basis of these notifications, we predict that there may be a balance between these mechanisms or processes. The counts of pairwise mutation (A>G and G>A, C>T and T>C, A>C and C>A, A>T and T>A, G>C and C>G, G>T and T>G) are similar. This is because for a specific biological sequence, the proportion of each base in the whole sequence will not vary too much even if mutations happen. We also analyze the unique mutations in **Figure 1B**. There are 23803 unique mutations. A>G owns the highest ratio, C>G owns the lowest ratio (5.43%=1293/23803), and SARS-CoV-2 also has the lowest ratio of C>G (1.25%) (Wang et al., 2020).

#### 3.1.2 Time analysis

**Figure 2:**
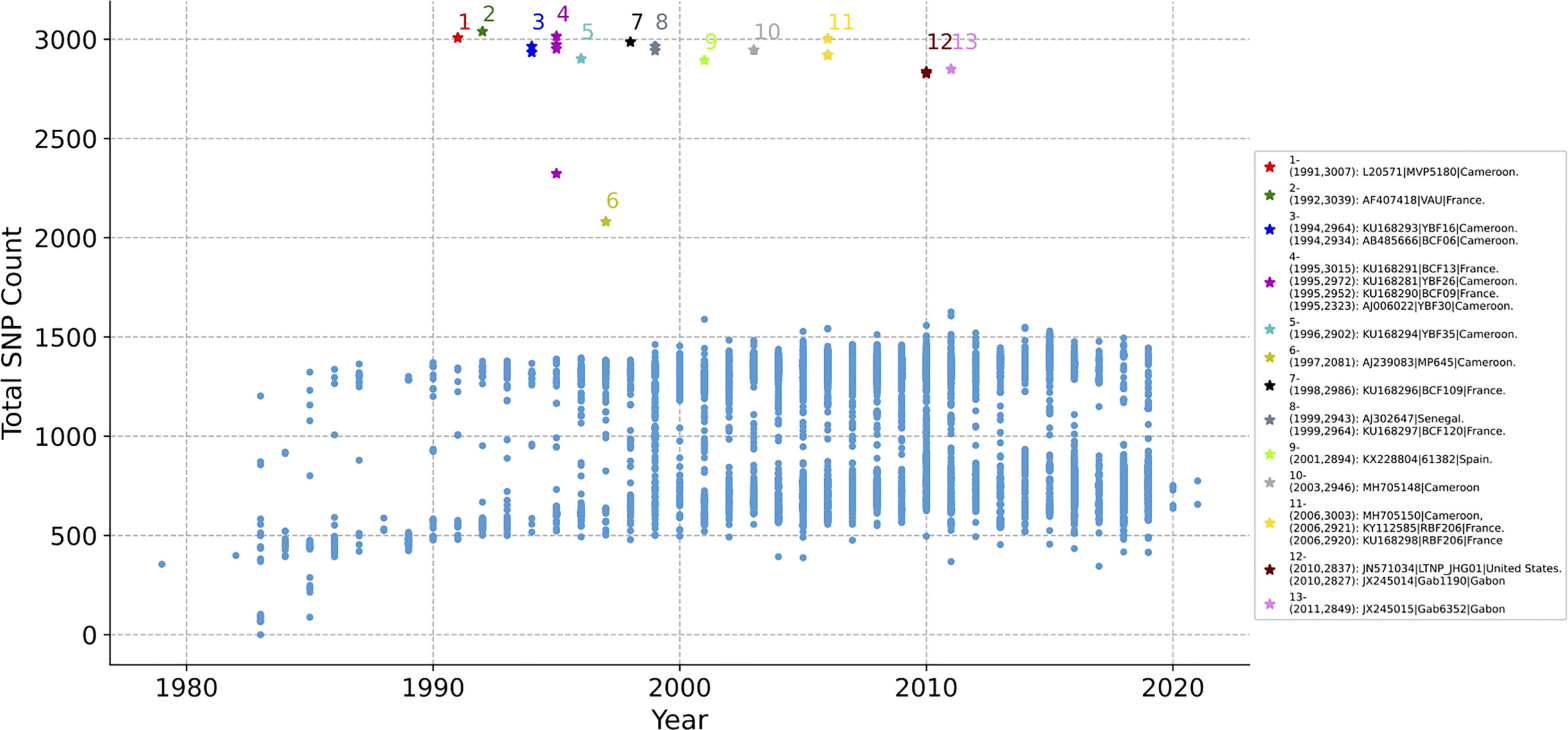
The distribution of SNP counts for each HIV-1 genome. Each genome with year label is aligned with the reference sequence of HIV-1, then the total SNP mutation of each genome is counted. Each dot represents a sequence. The total number of mutations in most sequences ranged from 500 to 1500 over time, but there are 21 genomes with abnormal mutation number, distributed in 13 years (from 1991 to 2011, marked with No. 1 to 13). No.- (Year, SNP Count): Accession Number| Patient Code| Country.

Whether the SNP of each HIV-1 genome changes over time is counted. The 11897 genomes are aligned with the reference sequence of HIV-1 first, and the non-unique single mutations of each genome is counted, as illustrated in **Figure 2**. The x-axis represents the year, y-axis represents total SNP count, and each dot represents a sequence. As time went on, the total number of mutations did not change significantly. The mutations in most genomes ranged from 500 to 1500, but 21 genomes have abnormal mutation numbers, which are distributed in 20 years (from 1991 to 2011, marked with No. 1 to No. 13). Of the 21 sequences, 9 are from Cameroon, 7 from France, 2 from Gabon, 1 from Senegal, 1 from Spain, and 1 from the United States. About half of these highly mutated sequences are located in African countries, but they are all distributed in Atlantic coastal countries (**Supplementary Figure 1**).

#### 3.1.3 Geographic analysis

**Figure 3:**
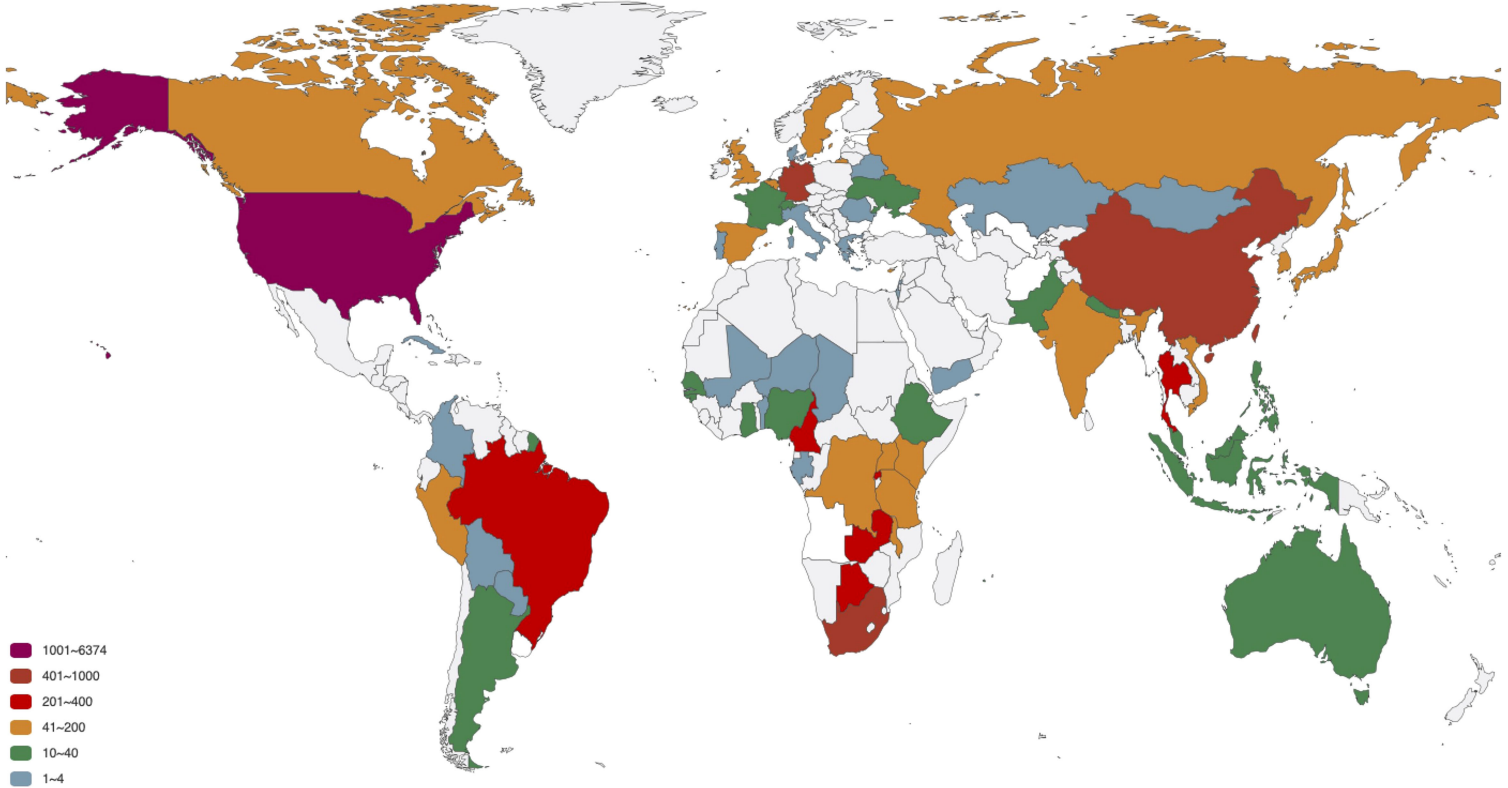
The number of HIV-1 complete genomes in each country. The 11897 sequences in our dataset are distributed in 78 countries.

The distribution of 12 SNP types in different countries is analyze. The 11897 sequences are distributed in 78 countries, as listed in **Supplementary Table S1**. To better exhibit the quantitative distribution of HIV-1 genome, we plot them in **Figure 3**. There are many HIV-1 cases in the Atlantic coastal countries, and the transmission may be related to traffic and living environment. The top 20 countries with larger number of genome include South Africa, which has the largest population of people with HIV of any country in the world (Ritchie and Max, 2018); South and South East Asia (For example, Thailand), which is the second most affected (UNAIDS,); United States, where over 675 thousand people died of AIDS since the beginning of the HIV epidemic (Prevention, C.f.D.C.a, 2016); Cameroon, where HIV-1 appears to have originated (Sharp and Hahn, 2011), etc. Among the 20 countries, 9 countries or regions belong to Africa (South Africa, Rwanda, Zambia, Botswana, Cameroon, Malawi, Kenya, Uganda, Tanzania); 3 countries belong to America (United States, Brazil, Canada); 5 countries belong to Europe (Germany, United Kingdom, Belgium, Sweden, Cyprus); 3 countries belong to Asia (China, Thailand, South Korea).

**Figure 4:**
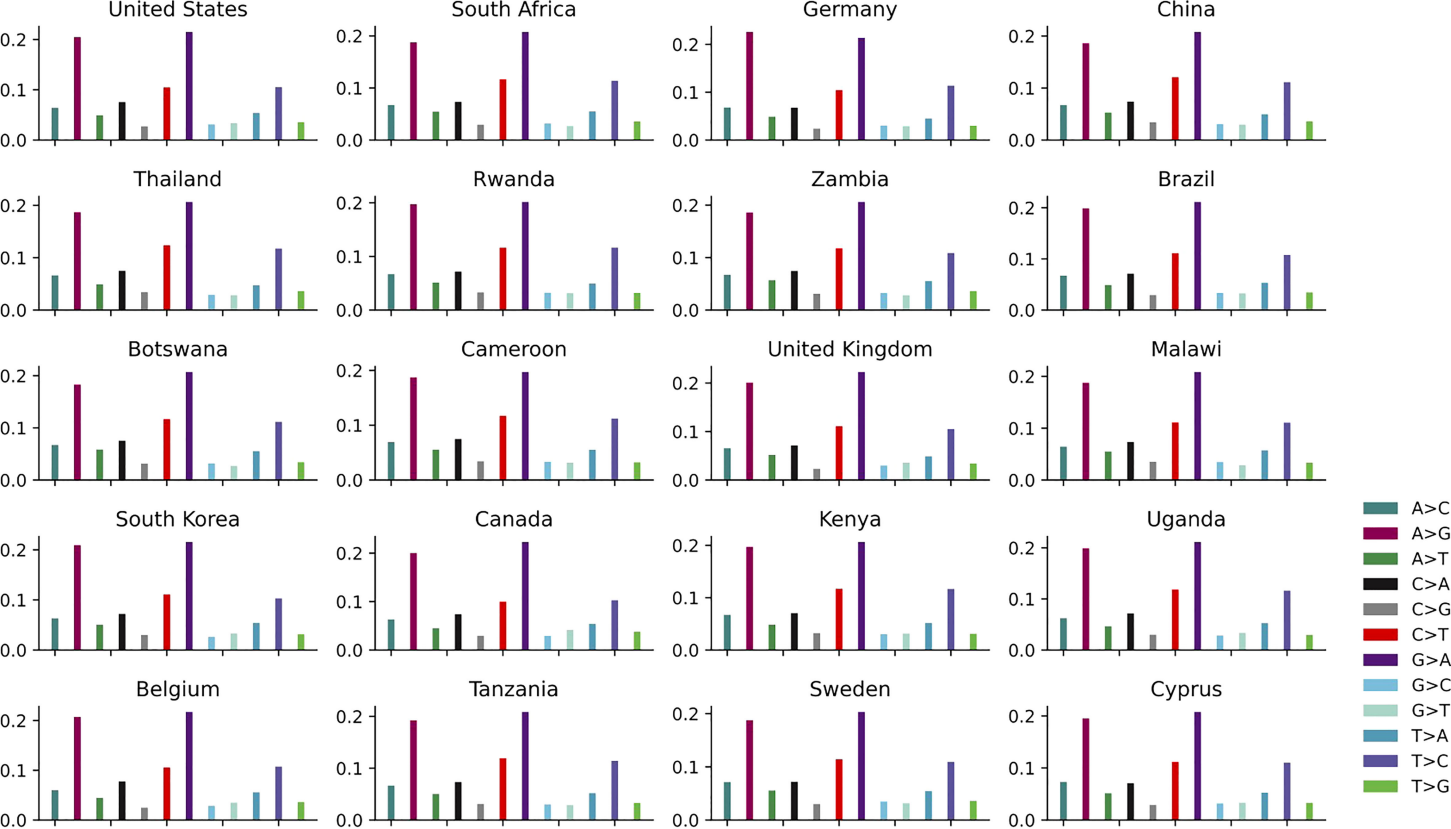
The distribution of 12 non-unique mutations in the top 20 countries with the larger number of genomes.

The distribution of 12 non-unique mutations in these top 20 countries is shown in **Figure 4** and **Supplementary Table S2**. The ratios of A>G, G>A, C>T and T>C are the top four highest among the 12 SNP types in each country, which is further convinced that these kinds of mutations are driven by RNA-APOBEC and RNA-ADAR editing. The APOBEC/ADAR protein will lead to C>T/A>G mutation and inhibit replication of HIV (Weiden et al., 2014), reducing the infectivity virus. The pairwise mutations (A>G and G>A, C>T and T>C, A>C and C>A, A>T and T>A, G>C and C>G, G>T and T>G) among these 20 countries also present similar counts. The distribution of 12 unique mutations is described in **Supplementary Figure S2**. The mutation A>G is relatively higher than G>A.

#### 3.1.4 Subtype analysis

The high mutations create many new HIV variants. During viral infection, a mutant genotype that is different from the infected genotype may be produced at any time. The proportion of 12 SNP types in different subtypes of HIV-1 is worth studied. HIV-1 is divided into a major group M (Taylor et al., 2008) and three minor groups O (D'Arc et al., 2015), N (Mourez et al., 2013) and possibly a group P (Plantier et al., 2009). The M group is subdivided further into subtypes: A, B, C, D, E, F, G, H, I, J, K, L (Hemelaar et al., 2006). There are also CRFs (circulating recombinant forms) from the recombination between different subtypes (Smith et al., 2005). For example, CRF01_AE is the recombination between subtypes A and E. Unique recombinant forms (URFs) are the viruses that have not been widely spread geographically (Fang et al., 2004). The study of the subtype diversity and high variability of HIV is of great significance for the diagnosis and treatment of AIDS. **Supplementary Figure S3 to S4 and Supplementary Table S3** elucidate the distribution of 12 unique and non-unique mutations in the top 20 subtypes with the larger number of genomes. The mutation A>G, G>A, C>T and T>C are more likely to occur than other mutations.

### 3.2 Mutation preferences on sequence context

There may be some interaction between nucleotides, so understanding point mutations in local regions of the genome is of great biological significance. The single nucleotide indicates A, C, G, T (also known as 1-mer). Dinucleotide sequences indicate AA, AC, AG, AT, CA, CC, CG, CT, GA, GC, GG, GT, TA, TC, TG, TT (also known as 2-mer). Trinucleotide sequences consist of 4^3^ sequence segments (also known as 3-mer). The k-mer (k=1, 2, 3, 4) counts are shown in **Supplementary Figure S5 to S8**. There are only 4 letters in the genetic code (A, C, G, T), so the probability of any 1-mer (or 2-mer or 3-mer or 4-mer) is $\frac{1}{4}$ ( $\frac{1}{16}$ or $\frac{1}{64}$ or $\frac{1}{256}$ , respectively), but there seems to be more k-mer containing A. Therefore, studying the local spelling of the virus genetic code may be able to recode its genomes, thereby reducing its infectivity, which is helpful to develop safe vaccines (Takata et al., 2017).

**Figure 5:**
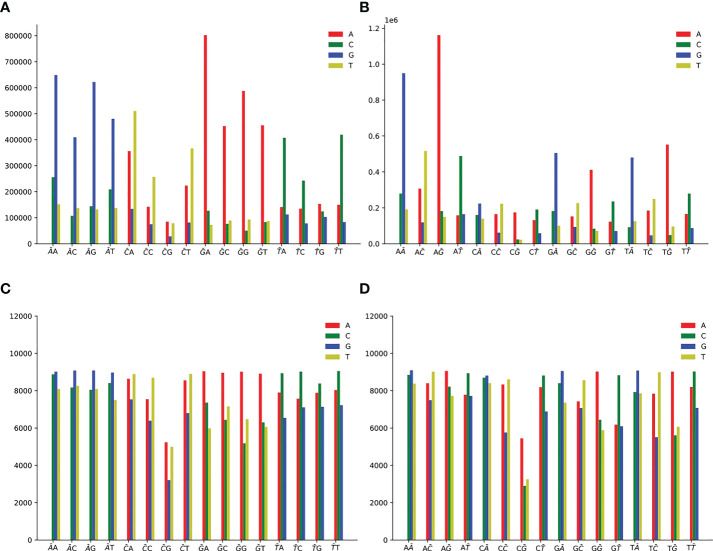
Single mutation counts of 2-mers. There are 16 dinucleotide sequences: AA, AC, AG, AT, CA, CC, CG, CT, GA, GC, GG, GT, TA, TC, TG, TT. **(A)** Single non-unique mutation counts at the first position of 2-mers. **(B)** Single non-unique mutation counts at the second position of 2-mers. **(C)** Single unique mutation counts at the first position of 2-mers. **(D)** Single unique mutation counts at the second position of 2-mers.

Single mutation counts of 2-mers are shown in **Figure 5**, and we observe the following mutation patterns:

- For non-unique mutation, there are 10,899,995 SNPs at the first position (**Figure 5A**), and 10,900,019 SNPs at the second position (**Figure 5B**).
- For unique mutation, there are 368,673 SNPs totally at the first position (**Figure 5C**), and 367,302 SNPs at the second position (**Figure 5D**).
- 2-mer AN or NA (N is one of A or C or G or T) for A>G mutation is the predominant context.
- 2-mer GN or NG for G>A mutation is the predominant context.
- 2-mer CH (H is one of A or C or T, the other degenerate base symbols can be found in **Supplementary Table 4**) or NC for C>T mutation is the predominant context.
- 2-mer TN or NT for T>C mutation is the predominant context.
- At the first position of CG, the count of C>A is slightly greater than that of C>T (**Figures 5A**, **C**). CG may have special significance. From **Supplementary Figure S6**, HIV-1 lacks CG sequence, and CG is less than other 2-mer. Due to evolutionary coincidence, the CG sequence will modify the letter C through chemical reaction, resulting in it being replaced by another letter. Therefore, the host may have some kind of cell monitoring system to recognize and destroy CG sequences, so as to prevent virus infection (Takata et al., 2017).

Single mutation counts of 3-mers are shown in **Supplementary Figure S9** and **Supplementary Figure S10**, and we observed the following mutation patterns:

- 3-mer ANN or NAN or NNA (N is one of A or C or G or T) for A>G mutation is the predominant context.
- 3-mer GNN or NGN or NNG for G>A mutation is the predominant context.
- 3-mer CMN, CTD (M is either A or C, D is one of A or G or T) or ACH (H is one of A or C or T) or DNC for C>T mutation is the predominant context.
- 3-mer THN, TGY (Y is either C or T) or WNT, CWT, GHT (W is either A or T) for T>C mutation is the predominant context.

Single mutation counts of 4-mers are shown in **Supplementary Figure S11 and Supplementary Figure S12**, and we could observe the stable mutation patterns: A>G, G>A, C>T and T>C.

### 3.3 Identification and validation of mutation preferences using k-mer natural vector

The sequence comparison of the mutants is necessary, and the method of comparing molecular sequences in above analysis is based on alignment. The other approach is based on alignment-free, which mainly include methods based on k-mer frequency (Sims et al., 2009), the length of common substrings (Leimeister and Morgenstern, 2014), graphical representation (Jeffrey, 1990), micro-alignments (Yi and Jin, 2013), and the number of word matches (Bromberg et al., 2016). Our team proposed k-mer natural vector to compare genomic sequences (Deng et al., 2011), which has been successfully applied to many classification and phylogenetic tasks (Sun et al., 2021; Sun et al., 2022a; Sun et al., 2022b). K-mer natural vector characterizes the statistical distribution of k-mers. Mutations can cause the changes in k-mers distributions. We take 1-mer as an example and give the definition of 1-mer natural vector. For the genomic sequence *S*=*s*_1_ *s*_2_ *s*_3_…*s*_*n*_ with length n, *L*={*A*,*C*,*G*,*T* *or* *U*} , the indicator functions are ${\text{w}}_{k}({\text{s}}_{i})=\{\begin{array}{c} 1,\;\;\;\;\;\;\text{if}\;{\text{s}}_{\text{i}}=\text{k}\;\\ 0,\;\;\;\;\text{otherwise} \end{array},$ and ${\text{w}}_{\text{k}l}({\text{s}}_{\text{i}})={\text{w}}_{\text{lk}}({\text{s}}_{\text{i}})=\{\begin{array}{c} 1,\;\;\;\;\;\;\text{if}\;{\text{s}}_{\text{i}}=\text{k}\;\text{or}\;\text{l}\;\\ 0,\;\;\;\;\;\text{otherwise}\;\;\; \end{array}$ , where *s*_*i*_,*k*,*l*∈*L*,*i*=1,2,3,…,*n*. The 1-mer natural vector with covariance component (1-mer NVC) is an 18-diemensional vector: $\left(n_{A},\;n_{C},\;n_{G},\;n_{T},\;\mu_{A},\;\mu_{C},\;\mu_{G},\;\mu_{T},D_{2}^{A},\;D_{2}^{C},\;D_{2}^{G},D_{2}^{T},\;Cov(A,\;C),\;Cov(A,\;G),\;Cov(A,\;T),\text{ Cov}(C,\;G),\;Cov(C,\;T),\;Cov(G,\;T)\right)$ , where *n*_*k*_ is the count of nucleotide k within sequence S: $n_{k}=\sum_{i=1}^{n}w_{k}(s_{i})$ , *μ*_*k*_ is the average location of nucleotide k within sequence S: $\mu_{k}=\sum_{i=1}^{n}\text{i}\frac{w_{k}(s_{i})}{n_{k}}$ , $D_{2}^{k}$ is the second central moment of positions of nucleotide k within sequence S: $D_{2}^{k}=\sum_{i=1}^{n}\frac{{\left(i-\mu_{k}\right)}^{2}w_{k}(s_{i})}{n_{k}n}$ , *Cov* (*k, l*) is the covariance between nucleotide k and nucleotide l within sequence S: $Cov(k,\;l)=\;\sum_{i=1}^{n}\frac{\left[i-\mu_{k}][i-\mu_{l}]w_{kl}(s_{i}\right)}{\text{n}\sqrt{n_{k}}\sqrt{n_{l}}}$ . Similarly, 2-mer natural vector with covariance component (2-mer NVC) characterizes the statistical features of 2-mers, which is a $\left(4^{k}\cdot 3+C_{4^{k}}^{2}\right)$ -dimensional vector:


$$\begin{array}{l} ({\text{n}}_{\text{AA}},{\text{n}}_{\text{AC}},n_{AG},n_{AT},n_{CA},n_{CC},n_{CG},n_{CT},n_{\text{GA}},{\text{n}}_{\text{GC}},{\text{n}}_{\text{GG}},{\text{n}}_{\text{GT}},{\text{n}}_{\text{TA}},{\text{n}}_{\text{TC}},{\text{n}}_{\text{TG}},{\text{n}}_{\text{TT}},\\ {\text{μ}}_{\text{AA}},{\text{μ}}_{\text{AC}},\mu_{AG},\mu_{AT},\mu_{CA},\mu_{CC},\mu_{CG},\mu_{CT},\mu_{\text{GA}},{\text{μ}}_{\text{GC}},{\text{μ}}_{\text{GG}},{\text{μ}}_{\text{GT}},{\text{μ}}_{\text{TA}},{\text{μ}}_{\text{TC}},{\text{μ}}_{\text{TG}},{\text{μ}}_{\text{TT}},\\ {\text{D}}_{2}^{\text{AA}},{\text{D}}_{2}^{\text{AC}},{\text{D}}_{2}^{\text{AG}},{\text{D}}_{2}^{\text{AT}},{\text{D}}_{2}^{\text{CA}},{\text{D}}_{2}^{\text{CC}},{\text{D}}_{2}^{\text{CG}},{\text{D}}_{2}^{\text{CT}},{\text{D}}_{2}^{\text{GA}},{\text{D}}_{2}^{\text{GC}},{\text{D}}_{2}^{\text{GG}},{\text{D}}_{2}^{\text{GT}},{\text{D}}_{2}^{\text{TA}},{\text{D}}_{2}^{\text{TC}},{\text{D}}_{2}^{\text{TG}},{\text{D}}_{2}^{\text{TT}},\\ \text{Cov}(\text{AA},\text{AC}),\text{Cov}(\text{AA},\text{AG}),\text{Cov}(\text{AA},\text{AT}),\text{Cov}(\text{AA},\text{CA}),\text{Cov}(\text{AA},\text{CC}),\ldots ,\text{Cov}(\text{TG},\text{TT})) \end{array}$$

**Figure 6:**
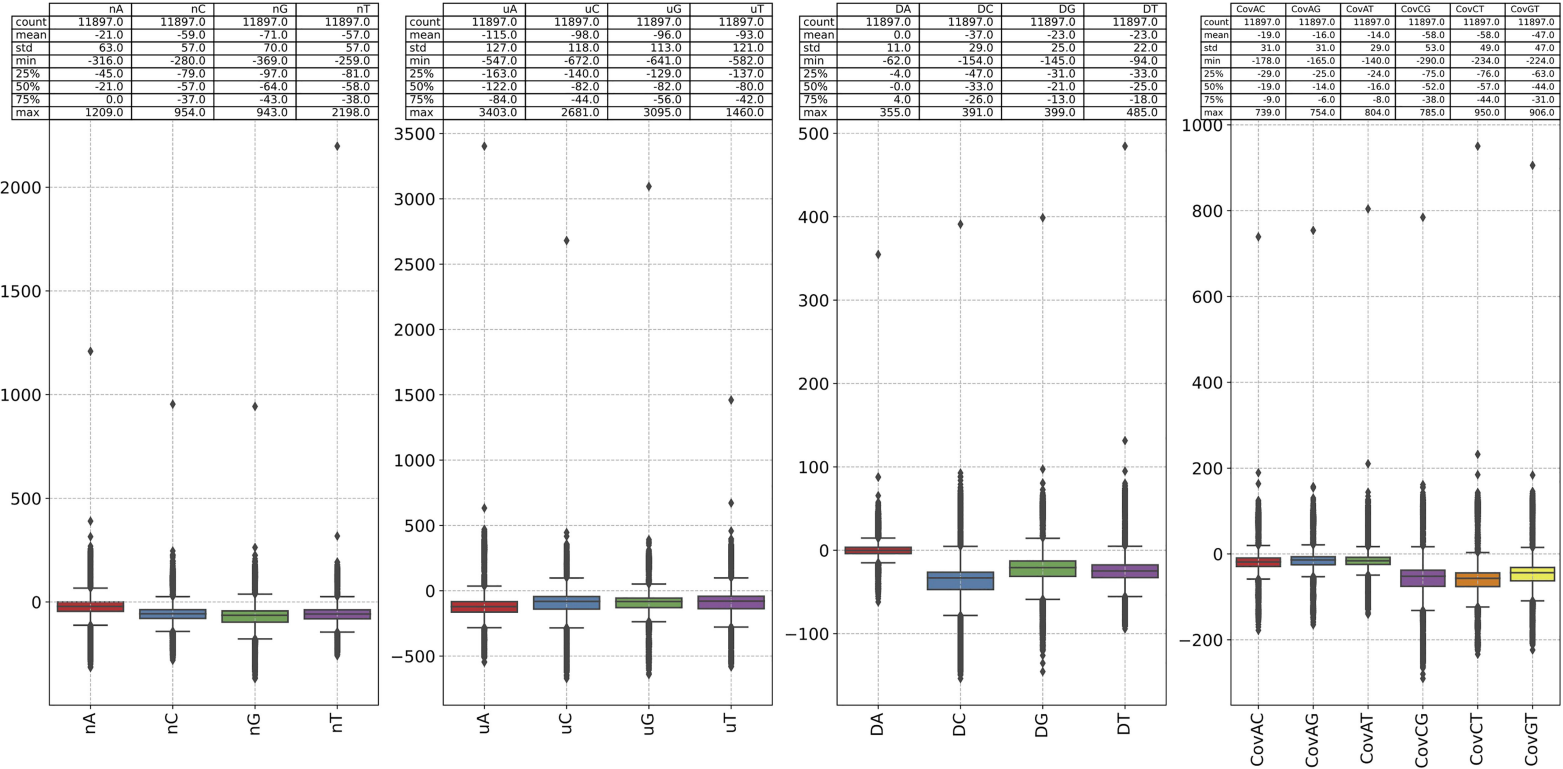
The difference between the 1-mer NVC of each sequence in our dataset and the 1-mer NVC of the reference sequence (NC_001802). The 1-mer NVC of the RefSeq is calculated first, and the 1-mer NVCs of the rest sequence subtracted it.

We use the alignment-free method, k-mer natural vector, to identify and validate the mutation preference conclusion obtained from the previous analysis. To achieve the goal, we first calculate the k-mer natural vector of HIV-1 RefSeq (NC_001802), and use the k-mer natural vectors of the rest sequences to subtract it. Mathematically, suppose sequence ***S***_1_ and sequence ***S***_2_ correspond to k-mer natural vectors ***NV***_1_ and ***NV***_2_ respectively, the nucleotide distribution difference (NDD) of the two sequences is described as NDD = ***NV***_2 –_ ***NV***_1_. K-mer natural vector can measure the changes of sequence context distribution and then identify the mutations in the genomes (Sun et al., 2022a). The changes results of k-mer (k=1, 2, 3) distribution are shown in **Figure 6**; **Supplementary Figure S13 and Supplementary Figure S14**. We observe the following k-mer distribution changing patterns:

- The change of count of 1-mer G is the largest (mean=-71, std=70), and C is second-ranked. The change of count of 1-mer A is minimum (mean=-21, std=63). The change of average location of 1-mer A is the largest (mean=-115, std=127), and C is second-ranked. The change of second central moment of 1-mer A is the minimum (mean=0, std=127). The change of covariance between 1-mer C and G is the largest (mean=-58, std=53).
- The change of count of 2-mer AG is the largest (mean=-35, std=27), and the change of count of 2-mer GA is the second largest (mean=-27, std=23). The average location of 2-mer CG grows the most (mean=156, std=361). The second central moment of 2-mer AA grows the most (mean=15), but the change is small (std=13). The change of second central moment of 1-mer CG is the largest (std=130). The change of covariance between 2-mer AA and CG is the largest (std=145).
- The change of count of 3-mer AGA is the largest (mean=-19, std=14), and the change of count of 3-mer GAG is the second largest (mean=-18, std=11). The count of 3-mer AAA grows the most. The changes of average location of 3-mer GCG is the largest (mean=600, std=639).

The results of **Figure 6**, **Supplementary Figure S13 and Supplementary Figure S14** give us reason to believe that the substitutions between nucleotide A and G, C and T are of great significance, and 2-mer CG may play an important role in viral infectivity. This analysis also shows the potential of natural vector in identifying the mutations in the genomes.

## 4 Discussion

### 4.1 Gene analysis

Furthermore, the ratios of 12 SNP types per gene are studied. HIV has nine major genes: Gag, Pol, Env, Tat, Rev, Nef, Vpr, Vpu, Vif. We extract each gene according to the gene location records of each genome in our dataset, then align the gene sequences with the gene sequence of reference sequence, and count the unique and non-unique mutations, as shown in **Table 1**. We can see that for each gene, mutation A>G has the highest ratio in unique case. In non-unique case, either A>G or G>A has the highest ratio among the 12 SNP types, which indicates the substitution of these two bases plays a vital role in viral infection.

**Table 1A:** The ratios of 12 unique mutations per gene.

Gene	A>C	A>G	A>T	C>A	C>G	C>T	G>A	G>C	G>T	T>A	T>C	T>G
*Vpu*	0.1265	**0.1320**	0.1238	0.0385	0.0385	0.0371	0.0908	0.0867	0.0853	0.0784	0.0812	0.0812
*Tat*	0.1060	**0.1160**	0.0989	0.0888	0.0831	0.0931	0.0802	0.0688	0.0616	0.0630	0.0745	0.0659
*Vpr*	0.1124	**0.1229**	0.1059	0.0614	0.0510	0.0706	0.1020	0.0784	0.0719	0.0680	0.0824	0.0732
*Rev*	0.1000	**0.1071**	0.0908	0.0796	0.0776	0.0806	0.1000	0.0929	0.0857	0.0561	0.0673	0.0622
*Vif*	0.1297	**0.1454**	0.1226	0.0584	0.0463	0.0684	0.0969	0.0663	0.0570	0.0592	0.0870	0.0627
*Nef*	0.1010	**0.1043**	0.1004	0.0697	0.0708	0.0724	0.0960	0.0933	0.0928	0.0659	0.0681	0.0653
*Gag*	0.1268	**0.1432**	0.1179	0.0618	0.0616	0.0716	0.0949	0.0724	0.0661	0.0568	0.0724	0.0544
*Env*	0.1164	**0.1206**	0.1147	0.0566	0.0570	0.0582	0.0835	0.0750	0.0753	0.0795	0.0833	0.0798
*Gag*-*Pol*	0.1348	**0.1631**	0.1197	0.0536	0.0454	0.0658	0.0975	0.0621	0.0572	0.0585	0.0861	0.0560

The bold values mean the mutation with the highest ratio. For each gene, mutation A>G has the highest ratio.

**Table 1B:** The ratios of 12 non-unique mutations per gene.

Gene	A>C	A>G	A>T	C>A	C>G	C>T	G>A	G>C	G>T	T>A	T>C	T>G
*Vpu*	0.0734	**0.1349**	0.1156	0.0955	0.0325	0.0938	0.1273	0.0298	0.0770	0.0716	0.0647	0.0839
*Tat*	0.1428	**0.1831**	0.0362	0.1342	0.0571	0.1044	0.1108	0.0355	0.0179	0.0368	0.1183	0.0229
*Vpr*	0.0702	0.1467	0.0219	0.0326	0.0137	0.1701	**0.2797**	0.0248	0.0235	0.0469	0.1329	0.0370
*Rev*	0.1008	**0.2157**	0.0382	0.1201	0.0343	0.1049	0.1606	0.0346	0.0273	0.0168	0.1094	0.0374
*Vif*	0.0734	0.1983	0.0291	0.0801	0.0227	0.1483	**0.2301**	0.0075	0.0288	0.0452	0.0952	0.0413
*Nef*	0.0427	0.1800	0.0690	0.0805	0.0398	0.1028	**0.2329**	0.0399	0.0301	0.0554	0.0803	0.0466
*Gag*	0.0707	**0.2179**	0.0332	0.0667	0.0252	0.1307	0.2169	0.0273	0.0221	0.0372	0.1247	0.0273
*Env*	0.0748	0.1843	0.0598	0.0849	0.0302	0.0947	**0.1913**	0.0422	0.0432	0.0662	0.0902	0.0381
*Gag*-*Pol*	0.0572	0.2337	0.0345	0.0557	0.0186	0.1278	**0.2394**	0.0189	0.0154	0.0380	0.1406	0.0203

The bold values mean the mutation with the highest ratio. For each gene, mutation A>G has the highest ratio.

### 4.2 Comparison of SIV mutations

HIV-1 is believed to originate in southern Cameroon (A country of West-central Africa) (Keele et al., 2006), and shows the high similarity with simian immunodeficiency virus (SIV, which infects wild chimpanzees or non-human primates) (Gao et al., 1999; Chahroudi et al., 2012). SIV has two types: SIVsmm in sooty mangabeys and SIVcpz in chimpanzees. A recent study on wild chimpanzee SIVcpz shows that infected chimpanzees would experience AIDS-like diseases. The late stage of SIV infection develops into SAIDS (Simian Acquired Immunodeficiency Syndrome), much like how HIV infection develops into AIDS. This inspires us to explore whether SIV and HIV-1 share the similar RNA editing behavior.

We download all 38 SIV complete genomes from https://www.hiv.lanl.gov/components/sequence/HIV/search/search.html, and regrad SIV ViralProj15501 as the reference (GenBank accession number: NC_001549, sequence length is 9623 base pairs). The structure comparison of reference sequences of HIV-1 and SIV can be found in **Supplementary Figure S15**. Their genome structures are similar, both viruses have Vif, Nef, Tat, Env, Gag-Pol genes. We present the distribution of 12 unique and non-unique mutations in **Supplementary Figure S16**. Mutation A>G is the highest ranked unique mutation, the reversed mutations G>A is the highest ranked non-unique mutation, which indicates the ADAR-editing may also play a crucial role in SIV infection.

### 4.3 Further discussion

In this research, we hypothesize that gene editing *via* APOBEC (C>T) and ADAR (A>G) is a driving force for RNA viral evolution (Wang et al., 2020), and focus on the mechanisms of HIV-1 from statistical perspective. Knowing how it works is very complicated, it depends more on the biological or clinical experiments. Our analysis gives a statistical basis for the effect study of ADAR gene on HIV replication. Many details are worth discussing. First, we download all data from the public HIV sequence Database, they are reliable. But there may exist sampling biases. For example, some relatively backward regions with AIDS cases do not have the perfect technologies for sequencing and sampling, which will result in a lack of data. The conclusion will be more persuasive if more data can be added. Moreover, if more labels are recorded (for example, virus source: blood or cell), the credibility of the results will be further improved. Second, we compare all sequences against HIV-1 RefSeq, it is a thoughtful choice. Literatures record that HIV-1 is a lentivirus discovered in 1983 (Barré-Sinoussi et al., 1983; Gallo et al., 1983). The HIV-1 RefSeq (Accession number is NC_001802) was sampled in France in 1983, which is a very early time. It is reasonable that the other genome sequences are aligned with HIV-1 RefSeq in the field of bioinformatics. Third, we only study the point mutation in local regions of the genome, but it will be of great significance to discuss the specific regions or the direction of the k-mer change count, which can be considered in future study.

## 5 Conclusions

We count and analyze 12 types of point mutations (A>C, A>G, A>T, C>A, C>G, C>T, G>A, G>C, G>T, T>A, T>C, T>G) from multiple perspectives. The global/geographical location/subtype/k-mer analysis results disclose that mutation A>G, G>A, C>T and T>C occupy the main superiority, which reveals the effectiveness of host APOBEC-editing and ADAR-editing. For non-unique mutations in population, the number of paired mutations (A>G and G>A, C>T and T>C, A>C and C>A, A>T and T>A, G>C and C>G, G>T and T>G) is similar. Time analysis displays that most genomes with abnormal mutation numbers are from African countries. In addition, our previously proposed method, k-mer natural vector, is applied to identify the k-mer distribution changing patterns in the genome. It is found that there is an important substitution pattern between nucleotides A and G, and 2-mer CG may be of very great significance for viral virulence.

## Data availability statement

The datasets presented in this study can be found in online repositories. The names of the repository/repositories and accession number(s) can be found in the article/**Supplementary Material**.

## Author contributions

SS-TY conceived the project and designed the study. NS collected data and carried out the data analysis including figures drawing and wrote the preliminary version of the paper. All authors have read and agreed to the published version of the manuscript.

## Funding

This work is supported by National Natural Science Foundation of China (NSFC) grant (12171275) and Tsinghua University Education Foundation fund (042202008).

## Acknowledgments

SS-TY is grateful to the National Center for Theoretical Sciences (NCTS) for providing an excellent research environment while part of this research was done.

## Conflict of interest

The authors declare that the research was conducted in the absence of any commercial or financial relationships that could be construed as a potential conflict of interest.

## Publisher’s note

All claims expressed in this article are solely those of the authors and do not necessarily represent those of their affiliated organizations, or those of the publisher, the editors and the reviewers. Any product that may be evaluated in this article, or claim that may be made by its manufacturer, is not guaranteed or endorsed by the publisher.
