# Supplementary material for: In-depth investigation of the point mutation pattern of HIV-1
Source: Front Cell Infect Microbiol. 2022 Nov 15;12:1033481. doi: 10.3389/fcimb.2022.1033481 (PMC9705751; doi:10.3389/fcimb.2022.1033481)
Supplement: Supplementary file 2 [file DataSheet_2.docx]

Supplementary Material

# Supplementary Figures and Tables

## Supplementary Figures

Supplementary Figure S1: Geographical distribution of 21 genomes with abnormal mutation numbers. Of the 21 sequences, 9 were from Cameroon, 7 from France, 2 from Gabon, 1 from Senegal, 1 from Spain, and 1 from the United States.

Supplementary Figure S2: The distribution of 12 unique mutations in the top 20 countries with the larger number of genomes.

Supplementary Figure S3: The distribution of 12 unique mutations in the top 20 subtypes with the larger number of genomes.

Supplementary Figure S4: The distribution of 12 non-unique mutations in the top 20 subtypes with the larger number of genomes.

Supplementary Figure S5: 1-mer statistics of our dataset.

Supplementary Figure S6: 2-mer statistics of our dataset.

Supplementary Figure S7: 3-mer statistics of our dataset.

Supplementary Figure S8: 4-mer statistics of our dataset.

A

B

C

Supplementary Figure S9: Single unique mutation counts of 3-mers. There are 64 segments of length 3: AAA, AAC, AAG, AAT, ACA, ACC, ACG, ACT, AGA, AGC, AGG, AGT, ATA, ATC, ATG, ATT, CAA, CAC, CAG, CAT, CCA, CCC, CCG, CCT, CGA, CGC, CGG, CGT, CTA, CTC, CTG, CTT, GAA, GAC, GAG, GAT, GCA, GCC, GCG, GCT, GGA, GGC, GGG, GGT, GTA, GTC, GTG, GTT, TAA, TAC, TAG, TAT, TCA, TCC, TCG, TCT, TGA, TGC, TGG, TGT, TTA, TTC, TTG, TTT. (A) Single unique mutation counts at the first position of 3-mers. (B) Single unique mutation counts at the second position of 3-mers. (C) Single unique mutation counts at the third position of 3-mers.

A

B

C

Supplementary Figure S10: Single non-unique mutation counts of 3-mers. There are 64 segments of length 3: AAA, AAC, AAG, AAT, ACA, ACC, ACG, ACT, AGA, AGC, AGG, AGT, ATA, ATC, ATG, ATT, CAA, CAC, CAG, CAT, CCA, CCC, CCG, CCT, CGA, CGC, CGG, CGT, CTA, CTC, CTG, CTT, GAA, GAC, GAG, GAT, GCA, GCC, GCG, GCT, GGA, GGC, GGG, GGT, GTA, GTC, GTG, GTT, TAA, TAC, TAG, TAT, TCA, TCC, TCG, TCT, TGA, TGC, TGG, TGT, TTA, TTC, TTG, TTT. (A) Single non-unique mutation counts at the first position of 3-mers. (B) Single non-unique mutation counts at the second position of 3-mers. (C) Single non-unique mutation counts at the third position of 3-mers.

Supplementary Figure S11: (A) Single unique mutation counts at the first position of 4-mers. There are $4^{4}=256$ segments of length 4.

Supplementary Figure S11: (B) Single unique mutation counts at the second position of 4-mers. There are $4^{4}=256$ segments of length 4.

Supplementary Figure S11: (C) Single unique mutation counts at the third position of 4-mers. There are $4^{4}=256$ segments of length 4.

Supplementary Figure S11: (D) Single unique mutation counts at the fourth position of 4-mers. There are $4^{4}=256$ segments of length 4.

Supplementary Figure S12: (A) Single non-unique mutation counts at the first position of 4-mers. There are $4^{4}=256$ segments of length 4.

Supplementary Figure S12: (B) Single non-unique mutation counts at the second position of 4-mers. There are $4^{4}=256$ segments of length 4.

Supplementary Figure S12: (C) Single non-unique mutation counts at the third position of 4-mers. There are $4^{4}=256$ segments of length 4.

Supplementary Figure S12: (D) Single non-unique mutation counts at the fourth position of 4-mers. There are $4^{4}=256$ segments of length 4.

Supplementary Figure S13: The difference between the 2-mer NVC of each sequence in our dataset and the 2-mer NVC of the reference sequence (NC_001802). The 2-mer NVC of the RefSeq was calculated first, and the 2-mer NVCs of the rest sequence subtracted it.

Supplementary Figure S14: The difference between the 3-mer NVC of each sequence in our dataset and the 3-mer NVC of the reference sequence (NC_001802). The 3-mer NVC of the RefSeq was calculated first, and the 3-mer NVCs of the rest sequence subtracted it.

A

| **Gene** | **Gene Length: bp** |
| --- | --- |
| vpu | 249 |
| tat | 261 |
| vpr | 291 |
| rev | 351 |
| vif | 579 |
| nef | 619 |
| gag | 1503 |
| env | 2571 |
| gag-pol | 4308 |

B

| **Gene** | **Gene Length: bp** |
| --- | --- |
| vpx | 357 |
| vif | 660 |
| nef | 672 |
| tat | 2457 |
| env | 2565 |
| rev1 | 2616 |
| gag-pol | 4418 |

Supplementary Figure S15: (A) Structure of the reference sequence of HIV-1 (NC_001802). The length of complete genome is 9181 bp, and HIV-1 has nine genes, including three structural genes—*Gag*, *Pol*, and *Env*; two regulatory genes—*Tat* and *Rev*; and four auxiliary genes—*Nef*, *Vpr*, *Vpu*, and *Vif*. (B) Structure of the reference sequence of SIV (NC_001549). The length of complete genome is 9623 bp, and SIV has seven genes: *Vpx*, *Vif*, *Nef*, *Tat*, *Env*, *Rev1*, *Gag*-*Pol*.

Supplementary Figure S16: (A) The distribution of 12 unique mutations counted from the 38 SIV aligned genomes. The unique mutation indicates that the same mutation in different genomes is only counted once. (B) The distribution of 12 non-unique mutations counted from the 38 SIV aligned genomes. The non-unique mutation indicates that the same mutation in different genomes is counted repeatedly.

## Supplementary Tables

Supplementary Table S1: The number of HIV-1 complete genomes in each country. The 11897 sequences in our dataset are distributed in 78 countries.

| **Order** | **Country** | **Count** | **Order** | **Country** | **Count** | **Order** | **Country** | **Count** |
| --- | --- | --- | --- | --- | --- | --- | --- | --- |
| 1 | United States | 6374 | 27 | Vietnam | 42 | 53 | Central African Republic | 4 |
| 2 | South Africa | 707 | 28 | France | 40 | 54 | Chad | 4 |
| 3 | Germany | 436 | 29 | Switzerland | 33 | 55 | Laos | 4 |
| 4 | China | 422 | 30 | Ghana | 29 | 56 | Paraguay | 4 |
| 5 | Thailand | 374 | 31 | Argentina | 28 | 57 | Cuba | 3 |
| 6 | Rwanda | 361 | 32 | Ethiopia | 25 | 58 | Denmark | 3 |
| 7 | Zambia | 354 | 33 | Nigeria | 19 | 59 | Gabon | 3 |
| 8 | Brazil | 228 | 34 | Senegal | 15 | 60 | Gambia | 3 |
| 9 | Botswana | 219 | 35 | Australia | 14 | 61 | Israel | 3 |
| 10 | Cameroon | 208 | 36 | Pakistan | 14 | 62 | Kazakhstan | 3 |
| 11 | United Kingdom | 180 | 37 | Nepal | 13 | 63 | Mongolia | 3 |
| 12 | Malawi | 170 | 38 | Ukraine | 12 | 64 | Niger | 3 |
| 13 | South Korea | 164 | 39 | Slovenia | 11 | 65 | Romania | 3 |
| 14 | Canada | 153 | 40 | Guinea-Bissau | 10 | 66 | Taiwan | 3 |
| 15 | Kenya | 145 | 41 | Indonesia | 10 | 67 | Yemen | 3 |
| 16 | Uganda | 145 | 42 | Malaysia | 10 | 68 | Bolivia | 2 |
| 17 | Belgium | 119 | 43 | Philippines | 10 | 69 | Colombia | 2 |
| 18 | Tanzania | 112 | 44 | Afghanistan | 9 | 70 | Georgia | 2 |
| 19 | Sweden | 108 | 45 | Myanmar | 9 | 71 | Mali | 2 |
| 20 | Cyprus | 97 | 46 | Uruguay | 9 | 72 | Belarus | 1 |
| 21 | Spain | 85 | 47 | Uzbekistan | 9 | 73 | Benin | 1 |
| 22 | India | 76 | 48 | Bulgaria | 8 | 74 | Dominican Republic | 1 |
| 23 | Japan | 51 | 49 | Cote Divoire | 8 | 75 | Greece | 1 |
| 24 | Peru | 49 | 50 | Angola | 6 | 76 | Italy | 1 |
| 25 | Dem Rep of Congo | 48 | 51 | Estonia | 6 | 77 | Macau | 1 |
| 26 | Russian Federation | 42 | 52 | Netherlands | 6 | 78 | Portugal | 1 |

Supplementary Table S2: The number of HIV-1 complete genomes in each country. The 11897 sequences in our dataset are distributed in 78 countries.

| **Order** | **Country** | **A>C** | **A>G** | **A>T** | **C>A** | **C>G** | **C>T** | **G>A** | **G>C** | **G>T** | **T>A** | **T>C** | **T>G** |
| --- | --- | --- | --- | --- | --- | --- | --- | --- | --- | --- | --- | --- | --- |
| 1 | United States | 298109 | 952920 | 229442 | 351649 | 126683 | 487795 | 1000642 | 144852 | 156391 | 250444 | 489784 | 166150 |
| 2 | South Africa | 63646 | 177769 | 51810 | 69448 | 27955 | 110433 | 196651 | 30229 | 25424 | 52184 | 107944 | 33923 |
| 3 | Germany | 21195 | 70401 | 15195 | 21046 | 7407 | 32496 | 66551 | 9468 | 9017 | 14050 | 35419 | 9301 |
| 4 | China | 35585 | 98748 | 28007 | 39209 | 18188 | 64185 | 110262 | 16181 | 15783 | 26313 | 59028 | 19115 |
| 5 | Thailand | 31528 | 88969 | 23361 | 35665 | 16250 | 58896 | 98321 | 13834 | 13530 | 22522 | 55917 | 17294 |
| 6 | Rwanda | 32858 | 96894 | 25174 | 35171 | 16175 | 57141 | 98974 | 15892 | 15482 | 24254 | 57179 | 15642 |
| 7 | Zambia | 32035 | 88772 | 27120 | 35577 | 14752 | 56094 | 98378 | 15493 | 13493 | 26369 | 51937 | 17330 |
| 8 | Brazil | 14239 | 42081 | 10356 | 15054 | 6194 | 23544 | 44707 | 7078 | 6916 | 11260 | 22794 | 7295 |
| 9 | Botswana | 20181 | 54977 | 17582 | 22652 | 9443 | 35079 | 62242 | 9622 | 8178 | 16646 | 33535 | 10277 |
| 10 | Cameroon | 20020 | 53980 | 16015 | 21599 | 9864 | 33772 | 56867 | 9543 | 9130 | 15959 | 32386 | 9317 |
| 11 | United Kingdom | 8233 | 25238 | 6513 | 8958 | 2886 | 13962 | 28031 | 3756 | 4500 | 6159 | 13235 | 4316 |
| 12 | Malawi | 14586 | 42482 | 12425 | 16635 | 7980 | 25164 | 47245 | 7863 | 6502 | 12981 | 25103 | 7618 |
| 13 | South Korea | 7408 | 24507 | 5904 | 8438 | 3526 | 13018 | 25249 | 3073 | 3875 | 6337 | 12064 | 3673 |
| 14 | Canada | 5850 | 18553 | 4156 | 6849 | 2738 | 9234 | 20652 | 2716 | 3846 | 4987 | 9519 | 3503 |
| 15 | Kenya | 12477 | 36579 | 8967 | 13082 | 6005 | 21708 | 38307 | 5601 | 5837 | 9567 | 21660 | 5772 |
| 16 | Uganda | 10441 | 33352 | 7792 | 12016 | 5028 | 19818 | 35441 | 4789 | 5649 | 8822 | 19480 | 4989 |
| 17 | Belgium | 5100 | 17649 | 3800 | 6613 | 2111 | 8989 | 18487 | 2428 | 2981 | 4747 | 9150 | 3073 |
| 18 | Tanzania | 9544 | 27615 | 7229 | 10534 | 4428 | 17132 | 29931 | 4342 | 4151 | 7449 | 16398 | 4754 |
| 19 | Sweden | 9478 | 24899 | 7361 | 9530 | 3966 | 15144 | 26941 | 4608 | 4175 | 7210 | 14491 | 4790 |
| 20 | Cyprus | 6508 | 17360 | 4575 | 6285 | 2568 | 9914 | 18442 | 2831 | 2961 | 4654 | 9786 | 2942 |

Supplementary Table S3: The number of HIV-1 complete genomes in the top 30 subtypes with the larger number of genomes.

| **Order** | **Subtype** | **Count** | **Order** | **Subtype** | **Count** |
| --- | --- | --- | --- | --- | --- |
| 1 | B | 6504 | 16 | F1 | 34 |
| 2 | C | 1646 | 17 | 08_BC | 33 |
| 3 | 01_AE | 519 | 18 | 07_BC | 26 |
| 4 | A1 | 433 | 19 | O | 22 |
| 5 | A1C | 114 | 20 | 01BC | 21 |
| 6 | 02_AG | 111 | 21 | A1CD | 20 |
| 7 | A1D | 102 | 22 | 20_BG | 19 |
| 8 | D | 84 | 23 | 22_01A1 | 17 |
| 9 | BF1 | 71 | 24 | 11_cpx | 16 |
| 10 | CD | 64 | 25 | U | 13 |
| 11 | 01B | 60 | 26 | 02A1 | 12 |
| 12 | BC | 56 | 27 | F2 | 10 |
| 13 | A6 | 52 | 28 | 85_BC | 10 |
| 14 | G | 35 | 29 | 35_A1D | 9 |
| 15 | 107 | 35 | 30 | 02A | 9 |

Supplementary Table S4: Degenerate base symbol.

| Symbol | R | Y | M | K | S | W | H | B | V | D | N |
| --- | --- | --- | --- | --- | --- | --- | --- | --- | --- | --- | --- |
| Bases represented | A/G | C/T | A/C | G/T | G/C | A/T | A/T/C | G/T/C | G/A/C | G/A/T | A/T/C/G |
